# GOT Care! 2.0: A Secondary Analysis of an Interprofessional Geriatric Care Model

**DOI:** 10.1093/geroni/igaf122.3163

**Published:** 2025-12-31

**Authors:** Millicent Malcolm, Catherine Rees, Greg Rhee, Anna-Rae Montano, Juliette Shellman

**Affiliations:** University of Connecticut, Storrs, Connecticut, United States; Middlesex Health, Middletown, Connecticut, United States; University of Connecticut School of Medicine, Farmington, Connecticut, United States; Hartford Hospital, Hartford, Connecticut, United States; University of Connecticut, Farmington, Connecticut, United States

## Abstract

Geriatric Outreach and Training with Care (GOT Care!) was created as a model for interprofessional geriatric education and practice. An interprofessional team of geriatric expert clinical faculty guided students from seven health professions in learning about and caring for chronically ill homebound older adults experiencing high emergency department (ED) use. The GOT Care! project was evaluated using the IHI Quadruple AIM framework and positive outcomes supported development of this model within a health system, however, gaps and need for further inquiry were also identified. GOT Care! 2.0, a secondary analysis of GOT Care! program data, was conducted with the aim of better understanding medical expenditures, healthcare utilization risk factors, and the observed reduction in ED visits for GOT Care! participants. Electronic health records, billing records and medical expenditures were extracted and analyzed for a 6 month pre- and post-program period. Direct costs were reduced significantly in the post-period (mean=$1,264.85) compared to the pre-period (mean=$4,457.44) (p < 0.001) and at 12 months (p < 0.001). Multiple factors associated with the likelihood of increased or decreased medical expenditures at 6 and 12 months were identified. With the support of the findings of the secondary analysis, an in-home interprofessional geriatric service pilot was launched. This pilot was based on the GOT Care! model and integrates the IHI Age-Friendly health system criteria focused on improving the IHI Quadruple AIM for a health system’s vulnerable older adult population.

